# The Modulation of the Pore Structure in Porous Carbon by Metal Salts and Its Application for Joining Silicon Carbide Ceramics

**DOI:** 10.3390/ma18102336

**Published:** 2025-05-17

**Authors:** Xishi Wu, Zehua Liu, Bingbing Pei, Haibo Wu, Zhengren Huang

**Affiliations:** 1 State Key Laboratory of Advanced Marine Materials, Ningbo Institute of Materials Technology and Engineering, Chinese Academy of Sciences, Ningbo 315201, China; liuzehua@nimte.ac.cn (Z.L.); peibingbing@nimte.ac.cn (B.P.); wuhaibo@nimte.ac.cn (H.W.); 2 Qianwan Institute of CNITECH, Qianwan New Area, Ningbo 315336, China

**Keywords:** porous carbon, pore structure control, polymerization-induced phase separation, iron ion, joining

## Abstract

In this work, the metal salts were introduced into the resin-solvent gel system to leverage their ortho-substitution effect, thereby accelerating the polymerization-induced phase separation process. Subsequent in-situ carbonization resulted in the preparation of porous carbon materials with three-dimensional interconnected pores. By precisely tuning the parameters of the resin-solvent-metal ion system, control over the pore structure of the porous carbon was achieved, with a porosity range of 16.5% to 66.5% and a pore diameter range of 8 to 248 nm. The addition of metallic salts can simply and effectively increase the pore structure after carbonization, making the infiltration of molten silicon easier. This is beneficial to the joining process of silicon carbide ceramics. Based on these findings, a high-reliability joining technique for large-sized (135 mm × 205 mm) silicon carbide ceramics was developed. The resulting interlayer was dense and defect-free, exhibiting a joining strength of 309 ± 33 MPa and a Weibull modulus of 10.67. These results highlight the critical role of structured porous media in advancing the field of large-sized ceramic joining.

## 1. Introduction

Porous carbons are functional materials with excellent properties, such as good pore structures, high porosity, good electrical conductivity, controllable pore sizes, and high specific surface areas [1,2,3,4]. Therefore, these materials are widely used in fields such as gas separation, water and air purification, adsorbent materials, catalyst carriers, chromatography, and the preparation of supercapacitors and carbide ceramics [5,6,7]. The application of porous carbon materials is closely related to their pore structure. Porous carbon materials are categorized into three types according to their pore sizes: micropores (<2 nm), mesopores (2~50 nm), and macropores (>50 nm) [8]. Micropores often function as catalytic active sites in specific energy conversion reactions, such as the oxygen reduction reaction (ORR). Mesopores enhance rapid mass transport and improve access to catalytic active sites, while macropores promote the swift diffusion of various molecules (e.g., oxygen) during the ORR process [9]. At the microstructural level, porous carbons exhibit diverse morphologies, including nanofiber films [10], nanospheres [11], activated carbon tubes [12], among others. Achieving superior performance requires rational control over the textural properties (pore volume, pore size distribution, degree of graphitization) and surface chemistry of carbon materials. This can be accomplished through strategies such as pore size regulation, channel optimization, doping modification, or catalyst loading, thereby fulfilling the specific demands of various applications. Fields such as supercapacitor fabrication [13] and gas chromatography require porous carbons in the mesoporous range (2–50 nm). Porous carbon materials with macroporous (>50 nm) structures are used in the preparation of new polymer catalysts, as conversion and separation materials, and to prepare carbide ceramics. Therefore, effectively controlling the pore structure of porous carbon materials is of great significance, especially for the preparation of carbide ceramics [14] Hucke [15] obtained a full-carbon porous prefab by pyrolyzing a polymer, and this prefab was reacted with molten silicon to obtain dense silicon carbide ceramics. This process can be extended to the joining of SiC ceramic with large size and complex shape [16,17]. By coating a layer of carbon containing porous preform on the surface of the parts to be joint, liquid silicon infiltrates the porous medium and reacts with carbon at high temperature, a SiC interlayer with near net size and high density can be obtained, which has great application potential. In this process, the pore structure of the porous carbon had a significant influence on the Si penetration process. Therefore, accurately controlling the pore structure of porous carbon materials is crucial for their successful application.

Currently, many methods have been used to prepare macroporous carbon materials, such as hard/soft template [18], activation [19], and polymerization-induced phase separation (PIPS) [20] methods. For instance, taking 100 nm-sized polystyrene nano-beads and polyvinylpyrrolidone as precursors, macroporous carbon microspheres (with macropore sizes of 50 to 90 nm) were obtained through a single-pot spray pyrolysis method [21].Silica spheres (about 180 nm) self-assembled into a polyacrylonitrile polymer shell, and the electrospinning-assisted hard template method was used to prepare mutually joining ordered macroporous hollow carbon nanofibers [22]. PIPS has many advantages compared to other methods, such as low manufacturing cost, simple process flow, and easy industrialization. Wang et al. [23] systematically studied the influence of process parameters on the use of a sterol resin to prepare a porous carbon pore structure. Xu et al. [20] reported that the control of the porous carbon pore structure was mainly related to the polymerization process of organic resins. Zhang Y. [24] and Yuan Z. [25,26] studied the influence of resin system composition and curing agent content on porous carbon pore structures by changing the mass ratio of furfuryl alcohol resin (FA) to phenolic resin (PF). When preparing porous carbon based on the PIPS method, the key to regulating the pore structure lies in the separation of the resin phase and the solvent phase. Some scholars have reported that divalent metal salts can effectively increase the degree of polymerization of phenolic resin [27], which is beneficial to phase separation. In our previous work [28], FeCl_2_ was added to a phenolic resin (PF) by the PIPS method to obtain a layered porous carbon material with meso-macro porous. The addition of FeCl_2_ changed the polymerization dynamics of the resin-solvent mixture, which affected the final porous carbon. At the same time [29], the adsorption properties of the prepared porous carbon material were studied. The adsorption capacity of this material for methylene orange (MO) reached 175.91 mg·g^−1^, demonstrating its potential as a magnetic separation superabsorbent candidate for the removal of MO and other dyes from wastewater.

In this paper, we continued our previous work and systematically studied the influence of thermodynamic factors (such as the organic resin/solvent mass ratio and solvent type of the metal ion-resin-solvent system) on the preparation of porous carbon pore structures. The influence of the Fe ion valence state (Fe^2+^, Fe^3+^) and coexisting anion types (NO_3_^−^, Cl^−^, etc.) on the porous carbon pore structure and phase was analyzed. The apparent porosity, bulk density, average pore size, and pore size distribution of the prepared porous carbon materials were characterized. The addition of metallic salts can simply and effectively increase the pore structure after carbonization, making the infiltration of molten silicon easier. This is beneficial to the joining process of silicon carbide ceramics. This work provides theoretical guidance for fully understanding the use of metal ions to regulate porous carbon pore structures. In addition, the porous carbon material prepared in this study has realized the high reliable connection of 135 mm × 205 mm large-scale SiC ceramic heat exchange components with complex pore structure.

## 2. Experimental Procedure

### 2.1. Materials

Phenol-formaldehyde resin (PF, industrial level, 99%) was purchased from FCP 15C, SIKA Tech, Lillesand, Norway. Ethyl alcohol (EG, ≥99%), diethylene glycol (DEG, ≥99%), triethylene glycol (TEG, ≥99%), polyethylene glycol 200 (PEG200, ≥99%), and polyethylene glycol 400 (PEG400, ≥99%) were purchased from Aladdin Chemistry Co., Ltd., Shanghai, China. Ferrous chloride (FeCl_2_, ≥99%), iron chloride (FeCl_3_, ≥99%), ferric nitrate (Fe(NO_3_)_3_, ≥99%), and ferrocene (C_10_H_10_Fe, ≥99%) were purchased from Sinopharm Chemical Reagent Co., Ltd., (Shanghai, China). The specimens for joining were SiC ceramics which were prepared by ourselves, which the 3-point flexure strength is over 400 MPa.

### 2.2. Porous Carbon Material Synthesis

The PF, organic solvent (one of EG, DEG, TEG, PEG200, PEG400), and corresponding metal salt (one of FeCl_2_, FeCl_3_, Fe(NO_3_)_3_, C_10_H_10_Fe) were mixed in a beaker and magnetically stirred for 30 min to ensure even mixing. The mixed solution was transferred into the crucible mold and subsequently placed in an oven for heating at 90 °C for 6 h. The temperature was then increased to 200 °C and maintained for 8 h. Finally, the crucible was removed and placed in a graphite furnace under the protection of an argon atmosphere and heated to 900 °C at a heating rate of 2 °C/min. The crucible was held at this temperature for 1 h and then cooled to room temperature to obtain the porous carbon material. In this work, variable parameters were employed as sample identifiers. Solvent variables were designated using the chemical abbreviations of the respective solvents (e.g., the ethylene glycol system is denoted as EG). The resin/solvent composition ratio was indicated by the mass ratio of resin to solvent (e.g., the PF/EG system with a ratio of 2:1 is labeled as 2:1). Samples containing metal salts were identified using the chemical formula of the corresponding salt (e.g., the FeCl_3_ system is marked as FeCl_3_). The blank control sample was designated as no–Fe.

### 2.3. Experimental Methods for Joining

In this work, the silicon carbide parts need to be ultrasonically cleaned in ethanol for 20 min before joining. The prepared resin based solution was coated between two silicon carbide ceramic parts to be connected, which were cured at 90–200 °C, pyrolyzed and carbonized at 900 °C, and infiltrated with molten silicon at 1600 °C after pickling, respectively, to realize the silicon carbide ceramic connection process.

### 2.4. Characterization

The phase compositions of the porous carbon samples were qualitatively or semi-quantitatively analyzed with an X-ray diffractometer (XRD, D/Max-2250V, Rigaku, Tokyo, Japan). The pore structures of the porous carbon samples were measured with a fully automatic mercury porosimeter (AutoPoreIV 9510, Norcross, GA, USA) to characterize their apparent porosity, bulk density, average pore size, and pore size distribution. Nitrogen adsorption-desorption experiments were performed with a fully automatic specific surface area and microporous pore analyzer (ASAP2020HD88, Norcross, GA, USA) to characterize the pore characteristics of the porous carbon samples. The weight and volume changes of the samples after curing and pyrolysis were evaluated by measuring the weight and volume of the cured body and the carbonized product. Fourier transform infrared spectroscopy (FT-IR) was performed on the cured body of the resin-solvent system with a Fourier transform infrared spectrometer (NICOLET Is10, Waltham, MA, USA). Sample microstructures were analyzed using a field emission scanning electron microscope (FE-SEM; SU-8220, Hitachi, Tokyo, Japan) equipped with an energy-dispersive X-ray spectrometer (EDS). A laser confocal Raman spectrometer (inVia, Renishaw, Gloucestershire, UK) was used to qualitatively and quantitatively analyze the molecular structures of the samples. The X-ray photoelectron spectroscopy (XPS) spectra were recorded on Kratos Ultra DLD XPS Kratos. The strength of the joining specimens was tested on an Instron 5566 testing system (Instron-5566, Boston, MA, USA). The internal structure of the connecting parts is tested by X-ray Industrial CT nondestructive testing equipment (450 kV). The carbon content was measured by LECO CS600 (LECO, Mönchengladbach, Germany), the iron content was quantified by ICP-OES (SPECTRO ARCOS II, Kleve, Germany), and the oxygen content was determined by a LECO ON836 (LECO, Mönchengladbach, Germany).

## 3. Results and Discussion

### 3.1. Influence of Solvent Type and Resin-Solvent Quality on Porous Carbon Pore Structure

Five organic solvents with different viscosities and molecular weights were selected to study the effects of organic solvent type on the porous carbon pore structure. Varying the solvent changed the viscosity of the resin-solvent system, affecting the thermodynamics of phase separation and regulating the pore structure. Table 1 lists the basic properties of the chosen solvents as well as the bulk density and apparent porosity of the porous carbon after carbonization. As the molecular weight of the solvent increased, the porosity gradually decreased and the bulk density gradually increased.

**Figure 1:**
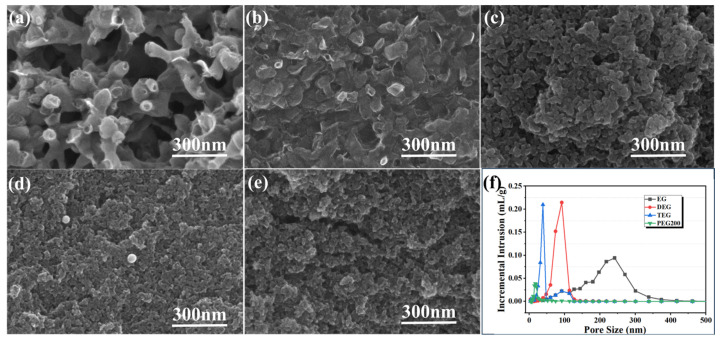
Effect of solvent type on the microstructure of porous carbon: (**a**) EG, (**b**) DEG, (**c**) TEG, (**d**) PEG200, and (**e**) PEG400, (**f**) mercury porosimetry pore size distributions.

SEM micrographs of the porous carbon samples obtained after the high-temperature carbonization of the resin-solvent systems prepared with different solvents are shown in Figure 1. The porous carbon materials exhibited continuous three-dimensional pore networks. The formation of these bi-continuous network structures can be explained by phase separation theory. The phase separation process of the phenolic resin-solvent system was carried out in accordance with the spinodal decomposition (SD) mechanism [30]: a micro-double continuous phase existed in the initial stage of phase separation, and a double continuous phase structure was eventually formed. After pyrolysis, due to the volatilization or decomposition of the alcohol, the obtained porous carbon samples exhibited a three-dimensional connected network-like structure. As the molecular weight of the solvent increased, the porosity gradually decreased, which was consistent with the measured porosity results shown in Table 1. This was because solvents with higher molecular weights increased the viscosity of the resin-alcohol system. Higher viscosity meant that it was more difficult for molecules to move during the reactive molecular phase separation process, reducing the rate of phase separation. After pyrolysis, the porosity of the porous carbon samples decreased and the pore size decreased. The pore size distribution of samples prepared in different solvents was characterized using the mercury porosimetry method (Figure 1f). The results indicated that the pore size progressively decreased with increasing solvent viscosity and molecular weight, which is in good agreement with the SEM observations. In addition to solvent viscosity and molecular weight, solvent polarity also plays a critical role in determining the state of monomers and products within the system, thereby influencing the polymerization process [31]. Higher solvent polarity enhances hydrogen bonding capabilities by enabling stronger hydrogen bonds with polar solutes, improving the solubility of resin monomers in the solvent, and facilitating the formation of homogeneous resin-solvent-metal salt systems. During resin gel polymerization, high-polarity solvents can retard the gelation process, extend the phase separation time window, allow for more extensive phase domain growth, and ultimately result in porous structures with larger pore diameters following high-temperature in-situ carbonization.

**Figure 2:**
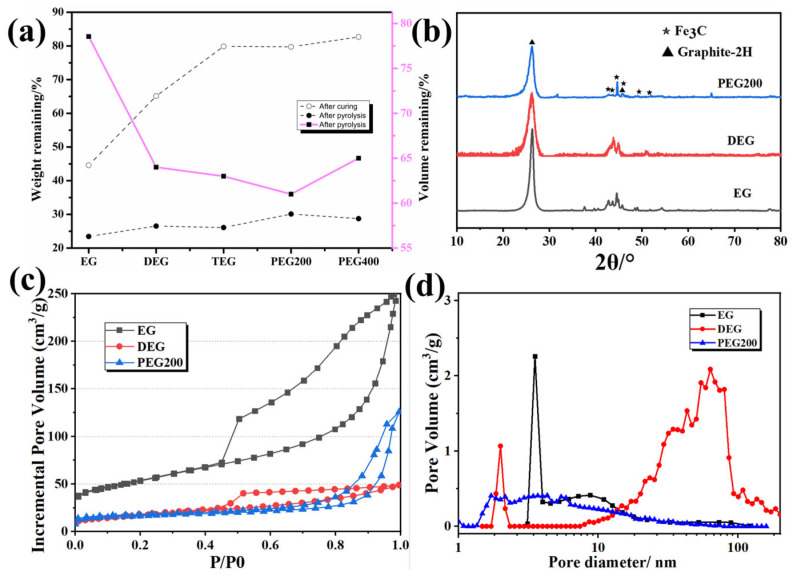
Effect of solvent type on (**a**) the weight and volume change of porous carbon samples after curing and pyrolysis, (**b**) XRD patterns, (**c**) nitrogen adsorption-desorption isotherms, and (**d**) nitrogen adsorption-desorption pore size distributions.

The changes in the remaining mass and volume of the resin-solvent solutions prepared using different types of solvents after curing and pyrolysis are shown in Figure 2. As the molecular weight and viscosity of the solvent increased, less mass was lost during the curing process. This was because the dispersed ethylene glycol-rich phase formed during the phase separation process increased. With increasing molecular weight, volume shrinkage became more severe after carbonization, which resulted in a lower porosity and smaller pore size. The crystal structure of the porous carbon materials after carbonization was characterized by XRD. As shown in Figure 2b, a sharp diffraction peak at 27.3° corresponds to the (002) plane of graphite carbon, while the prominent peaks at 37.6°, 43.7°, and 54.4° are attributed to the (210), (102), and (230) planes of orthorhombic Fe_3_C [32]. These results confirm the coexistence of graphite carbon and orthorhombic Fe_3_C in the porous carbon material. The peak positions and peak shapes of the porous carbons prepared by different solvents are consistent, suggesting that the type of solvent has no significant effect on the phase composition of the porous carbon. Figure 2c displays the nitrogen adsorption isotherms of different solvent samples, all of which exhibit behaviors characteristic of type IV curves with H4-type hysteresis loops. The specific surface areas of EG, DEG, and PEG200, as determined by the Brunauer-Emmett-Teller (BET) method, are 188, 61, and 54 m^2^·g^−1^, respectively. As the molecular weight of the solvent increases, the specific surface area decreases gradually, likely due to a reduction in porosity. The mesopore size distribution is illustrated in Figure 2d, with pore diameters predominantly ranging from 2–4 nm. The formation of mesopores is primarily attributed to the Fe-catalyzed C–H_2_O reaction [29].

**Figure 3:**
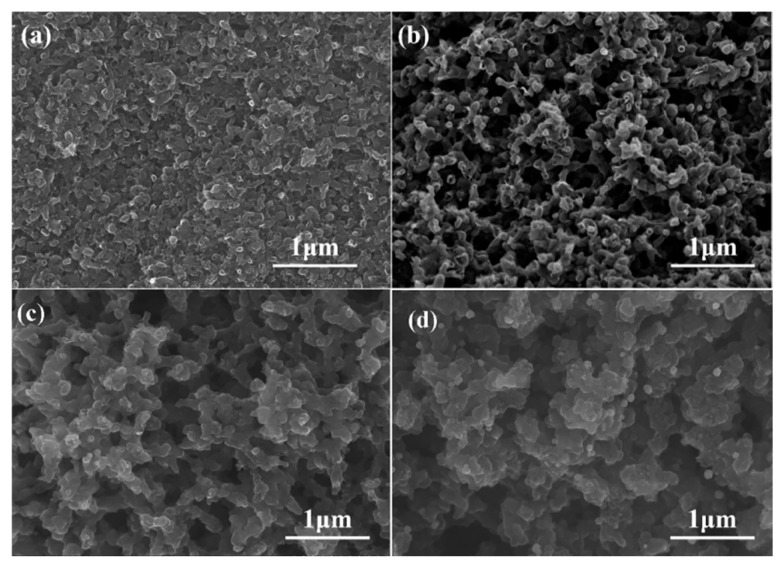
Morphologies of carbonized products prepared from resin mixtures with different PF/EG ratios: (**a**) 7:3, (**b**) 5:5, (**c**) 1:2, and (**d**) 2:8.

Based on the obtained results on the effect of solvent type, ethylene glycol was selected as the organic solvent to study the influence of PF/EG quality on the porous carbon pore structure. The microstructures of carbonized products prepared from resin mixtures with different PF/EG mass ratios were evaluated by SEM, as shown in Figure 3. Changing the PF/EG mass ratio significantly affected the pore structures of the porous carbon samples. With increasing PF/EG mass ratio (that is, with increasing ethylene glycol content in the resin-solvent system), smaller pores were formed and the carbon skeleton became finer. When the PF/EG mass ratio was >1 (ratio of 7:3, as shown in Figure 3a), the ethylene glycol content was lower than the resin content. At this ratio, the porous carbon structure was mainly based on the carbon matrix, and the pores tended to be isolated. This showed that with a small amount of ethylene glycol, the solvent-rich phase formed after the resin-solvent system phase separation process did not exist as a continuous phase. As the PF/EG mass ratio further decreased (ratios of 5:5 and 1:2, as shown in Figure 3b,c), the carbon matrix skeleton became thinner. This was because the larger amount of ethylene glycol meant it was easier for the solvent-rich phase to form a continuous phase, enabling the formation of a double continuous network pore structure after pyrolysis. However, when the ethylene glycol content was too high (ratio of 2:8, as shown in Figure 3d), carbon skeleton formation was not possible, causing the sample to shatter after pyrolysis.

The apparent porosities and bulk densities of the carbonized products prepared from resin mixtures with different PF/EG mass ratios are listed in Table 2. With increasing ethylene glycol content, the porosity significantly increased, the bulk density gradually decreased, and the residual carbon rate gradually decreased. This was because as the amount of ethylene glycol increased, more substances decomposed and volatilized during pyrolysis, resulting in larger pores and a smaller residual mass.

**Figure 4:**
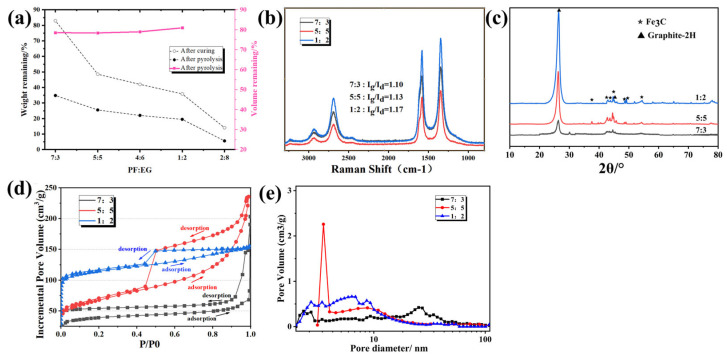
Effect of PF/EG mass ratio on (**a**) the weight and volume change of samples after curing and pyrolysis, (**b**) raman spectra, (**c**) XRD patterns, (**d**) nitrogen adsorption-desorption isotherms, and (**e**) nitrogen adsorption-desorption pore size distributions.

The changes in the remaining mass and volume of porous carbon samples prepared using resin-solvent solutions with different PF/EG mass ratios after curing and pyrolysis (Figure 4). With increasing ethylene glycol content, the quality loss during the curing process gradually increased. After curing and carbonization treatment, the remaining quality (residual carbon rate) gradually decreased with increasing ethylene glycol content. This trend was consistent with the results shown in Figure 3 and Table 2.

The Raman spectra of the three porous carbon samples are shown in Figure 4b. The intensity ratio (Ig/Id) of the D-peak and G-peak is commonly used to characterize the degree of graphitization of carbon materials [33], and a greater Ig/Id ratio indicates a higher graphitization degree. The results indicated that as the resin/solvent ratio decreased, the degree of graphitization increased. This could be attributed to the reduced residual carbon mass, which enhances the effective concentration of metal salt catalysts. Metal salts are known to facilitate the graphitization of carbon. The XRD results (Figure 4c) indicated that the primary crystal structures in the porous carbon were graphite carbon and Fe_3_C. As the resin-to-solvent mass ratio increases, the intensity of the diffraction peaks became stronger while the full width at half maximum (FWHM) decreases, suggesting an enhancement in crystallinity. This observation was consistent with the Raman spectroscopy results. The mesopore size distribution of the 5:5 and 1:2 samples is illustrated in Figure 2d, with pore diameters predominantly ranging from 2 to 4 nm. The formation of these mesopores is primarily attributed to the iron-catalyzed C–H_2_O reaction. According to SEM observations, the dominant pore structures of the 5:5 and 1:2 samples are in the macropore range. For the 7:3 sample, the mesopore size distribution spans two ranges: 2–4 nm and 20–40 nm. The formation of pores in the 20–40 nm range is ascribed to the low solvent content, which induces polymerization-driven phase separation and the development of smaller solvent-rich domains. These domains eventually lead to the formation of a finer pore structure after carbonization.

### 3.2. Influence of Fe Ion Valence State on Pore Structure

**Figure 5:**
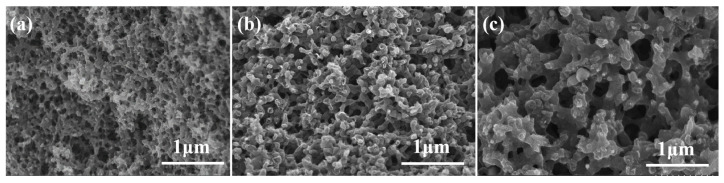
SEM images showing the microstructure of porous carbon samples prepared with (**a**) no Fe, (**b**) FeCl_2_, and (**c**) FeCl_3_.

The microstructures of the porous carbon samples obtained after carbonization of three resin mixtures prepared without the addition of metal ions (no Fe), with FeCl_2_, and with FeCl_3_ are shown in Figure 5. After adding FeCl_2_ and FeCl_3_ to the resin-solvent system, a three-dimensional connected pore structure was obtained after carbonization. Moreover, the pore size was significantly enhanced compared with the sample prepared without the addition of metal ions. Compared with the addition of FeCl_2_, the pores of the porous carbon sample prepared with FeCl_3_ were larger in size and were more widely distributed. However, the sample prepared using FeCl_3_ had a less uniform pore structure.

The specific structural parameters of the porous carbon samples prepared using no Fe, FeCl_2_, and FeCl_3_ were analyzed, as shown in Table 3. Compared with the sample prepared without using Fe, the porosity and average pore size were significantly increased by the addition of Fe ions, while the bulk density was greatly reduced. Therefore, the addition of metal ions was conducive to pore formation [34]. When FeCl_2_ was added, the average pore size of the resulting porous carbon was 190 ± 15 nm, the apparent porosity was 63.3 ± 1.7%, and the bulk density was 0.73 g·cm^−3^. In contrast, the porous carbon sample prepared with Fe^3+^ had a slightly larger average pore size of 248 ± 29 nm, a lower apparent porosity of 50.9 ± 1.3%, and a higher bulk density of 0.95 g·cm^−3^.

**Figure 6:**
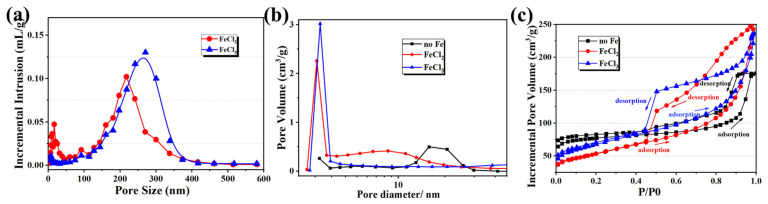
Textural analysis of porous carbon samples prepared with no Fe, with FeCl_2_, and with FeCl_3_: (**a**) mercury porosimetry pore size distributions, (**b**) nitrogen adsorption-desorption pore size distributions, and (**c**) nitrogen adsorption-desorption isotherms.

The pore size distributions of the samples were evaluated with mercury porosimetry, as shown in Figure 6a. The porous carbon obtained using FeCl_2_ had a pore size distribution of 150~300 nm. The sample prepared with Fe^3+^ had a wider pore size distribution of 120~400 nm, which was consistent with the SEM images shown in Figure 5. Mercury porosimetry is mainly used to evaluate pores with diameters >50 nm. Therefore, nitrogen adsorption-desorption tests were used to analyze the micro-mesoporous structures of the porous carbon materials, as shown in Figure 6b,c. The porous carbon prepared without Fe had a 10~30 nm pore size distribution. The porous carbon samples prepared using FeCl_2_ and FeCl_3_ both showed pores concentrated around 3.5 nm. However, the sample prepared with FeCl_2_ had a broader mesoporous pore size distribution of 4~20 nm, while the pore size distribution of the sample prepared with FeCl_3_ was much narrower. The FeCl_3_ has a higher Lewis acidity than FeCl_2_ and has the coordination ability with the oxygen-containing functional groups of carbon precursors. In the Fe^3^⁺ system, the metal-organic complex formed by strong coordination can induce a more stable double continuous phase structure, and a wider distribution pore structure network is formed after carbonization. As shown in Figure 6c, the adsorption-desorption isotherms of the samples of no Fe, FeCl_2_, and FeCl_3_ fit the type IV adsorption isotherms, because the adsorption isotherms at higher relative pressures P/P0 (0.7 < P/P0 < 1.0) showed clear hysteresis loop, which is also typical of the adsorption isotherm of mesoporous structure.

**Figure 7:**
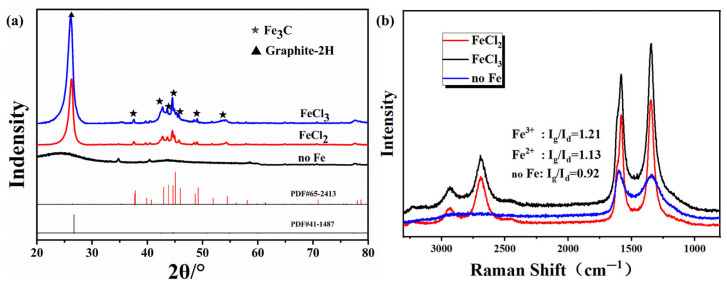
(**a**) XRD patterns and (**b**) Raman spectra of the porous carbon samples prepared using no Fe, with FeCl_2_, or with FeCl_3_.

The XRD patterns of the porous carbon samples (no Fe, FeCl_2_, and FeCl_3_) are shown in Figure 7a. The diffraction pattern of the porous carbon (no Fe) only showed a gentle amorphous peak at about 23°, indicating that an amorphous carbon structure was obtained. The FeCl_2_ sample exhibits a sharp diffraction peak at 27°, corresponding to the (002) plane of graphite carbon. The prominent peaks at 37.6°, 43.7°, and 54.4° are attributed to the (210), (102), and (230) planes of orthorhombic Fe_3_C, respectively. After introducing FeCl_3_, the peak positions remain essentially unchanged compared with the FeCl_2_ sample, suggesting that regardless of the valence state of the added metal ions, they predominantly exist in the form of Fe_3_C after carbonization. Notably, compared with the FeCl_2_ sample, the FeCl_3_ sample demonstrates a significantly enhanced intensity of the graphite peak and a reduced full width at half maximum (FWHM), indicating an improvement in the degree of graphitization. The graphitization process of carbon materials is significantly influenced by the catalytic effect of metal ions, the essence of which lies in the directional induction ability of transition metal ions on the reconstruction of the sp^2^ hybridization network during the pyrolysis of the carbon matrix. Fe^3^⁺ has a higher charge density compared to Fe^2^⁺, and this characteristic enhances its coordination with the oxygen-containing functional groups of the carbon precursor, forming a more stable metal-carbon intermediate phase during the carbonization stage. This coordination stabilization effect effectively reduces the activation energy barrier for the growth of graphite microcrystals. The Raman spectra of the three porous carbon samples are shown in Figure 7b. The porous carbon sample prepared with the addition of FeCl_2_ had an Ig/Id ratio of 1.13, while that of the porous carbon obtained with the addition of FeCl_3_ increased to 1.19. Therefore, FeCl_3_ was more conducive to enhancing the degree of graphitization than FeCl_2_, which was consistent with the XRD results.

**Figure 8:**
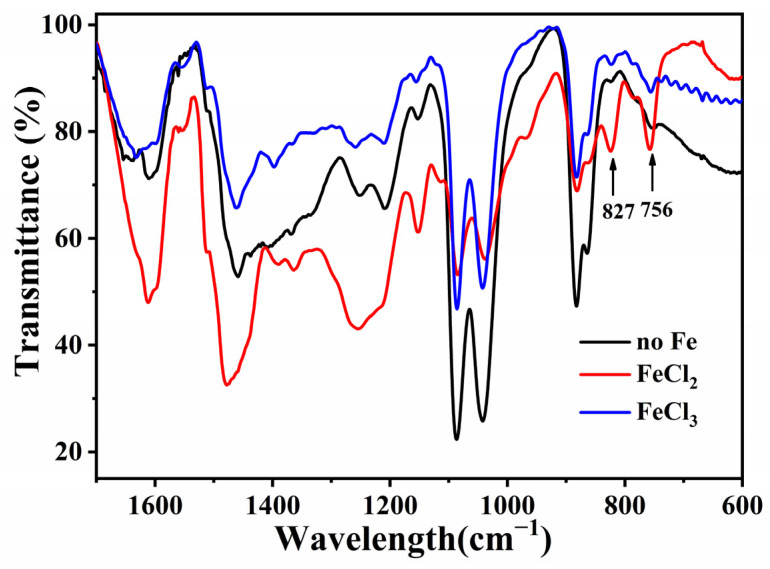
FT-IR spectra of resin mixtures prepared without Fe and with the addition of FeCl_2_ or FeCl_3_ after pre-curing at 90 °C.

The FT-IR spectra of the resin mixtures prepared without Fe and with the addition of FeCl_2_ or FeCl_3_ after pre-curing at 90 °C are shown in Figure 8. After adding FeCl_2_, the resin mixture showed strong absorption peaks at 827 and 750 cm^−1^. An absorption peak at 825 cm^−1^ corresponds to the bending vibration of the C–H bonds of 1,4-disubstituted and 1,2,4- trisubstituted benzene rings [35], indicating the existence of para-substituted phenol groups. An absorption peak at 756 cm^−1^ corresponds to the bending vibration of the C–H bonds of 1,2-disubstituted and 1,2,6 trisubstituted benzene rings [35], indicating the existence of ortho-substituted phenol groups. In the phenolic structure of phenolic resin, the ortho position exhibits the lowest reactivity, whereas the para position demonstrates the highest reactivity. Consequently, during the polymerization reaction involving the phenolic structure, the –CH_2_OH group preferentially undergoes addition at the para position, leaving the less reactive ortho position, which becomes more challenging for subsequent addition reactions. Upon introducing metal ions, their directing effect facilitates ortho substitution of the –CH_2_OH group, thereby preserving the highly reactive para position. This enables easier reactions with the –CH_2_OH group or other polymer molecules in subsequent polymerization steps, ultimately yielding a polymer product with a higher degree of polymerization. As the curing extent of the resin mixture increases, the phase separation between the resin and solvent accelerates, promoting the formation of pore structures. Unlike the resin prepared with the addition of FeCl_2_, the resin mixture prepared with the addition of FeCl_3_ did not show strong absorption peaks at 827 and 750 cm^−1^. Therefore, FeCl_3_ only acted as a catalyst during the polymerization reaction and was not involved in the formation of the polymer skeleton. This explained the lower porosity of the porous carbon sample prepared with FeCl_3_ compared with that of the sample prepared with FeCl_2_.

### 3.3. Influence of Coexisting Anion Type on Porous Carbon Pore Structure

**Figure 9:**
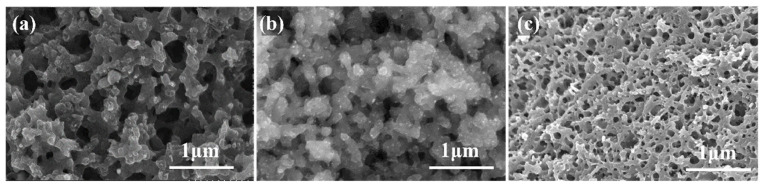
SEM images showing the microstructures of porous carbons prepared with (**a**) FeCl_3_, (**b**) Fe(NO_3_)_3_, and (**c**) C_10_H_10_Fe.

Porous carbon samples were obtained by pyrolyzing resin mixtures separately prepared with the addition of FeCl_3_, Fe(NO_3_)_3_, or C_10_H_10_Fe. The microstructures of these samples were evaluated by SEM, as shown in Figure 9. The pore structures of the samples prepared using FeCl_3_, and Fe(NO_3_)_3_ were similar, with three-dimensional pore networks. The porous carbon sample prepared with the addition of ferrocene was similar to the porous carbon sample prepared without the addition of Fe, with a small pore size and low pore structural uniformity.

**Figure 10:**
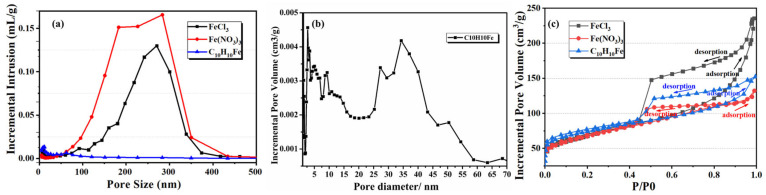
Pore size distributions (**a**,**b**) and N_2_-siotherm features (**c**) of porous carbon samples prepared with different anions.

The pore structure parameters of the porous carbon samples prepared with different anions were evaluated by mercury porosimetry and nitrogen adsorption-desorption, as shown in Table 4. The pore size distributions obtained from the nitrogen adsorption- desorption data are shown in Figure 10. The porous carbon prepared with Fe(NO_3_)_3_ had an average pore size of 228 ± 21 nm, an apparent porosity of 51.4 ± 1.9%, a bulk density of 0.97 g·cm^−3^, and a pore size distribution in the range of 100~350 nm. This was similar to the porous carbon sample prepared with the addition of FeCl_3_. The porous carbon sample prepared with C_10_H_10_Fe had an average pore size of 8 ± 3 nm, an apparent porosity of 30.9 ± 1.9%, a bulk density of 1.21 g·cm^−3^, and a pore size distribution of 2~40 nm. As shown in Figure 10c, the adsorption-desorption isotherms of the three samples were all type IV (the typical isotherm type of mesoporous structures).

In the preparation system of porous carbon materials, the anionic coordination environment has a significant regulatory effect on the thermal decomposition kinetics of metal ion and the sol-gel phase separation process. As strong electrolyte salts, FeCl_3_ and Fe(NO_3_)_3_ show excellent dissociation characteristics in glycol solvent. Through the coordination between metal cations and resin monomers, the topological growth of three-dimensional network structure can be effectively induced, so as to retain the macro through multi-stage pore structure in the process of pyrolysis and carbonization. In contrast, metal organic compound C_10_H_10_Fe is limited by the coordination saturation effect, and it is difficult to achieve effective dissociation in polar solvent system. Due to the coordination inertia of the Fe centers in the molecules, the metal catalytic sites were not exposed enough and could not effectively participate in the template guiding role in the process of resin polymerization. This limited metal organic synergistic effect directly inhibits the formation dynamics of bicontinuous phases in the process of phase separation, and ultimately leads to the disordered development of pore structure. It is worth noting that at the high temperature carbonization stage, the Fe species in C_10_H_10_Fe molecule can still be partially transformed into nano catalytic sites, which catalyzes the surface reaction between carbon matrix and gasification medium, forming pores of about 3.5 nm [36].

**Figure 11:**
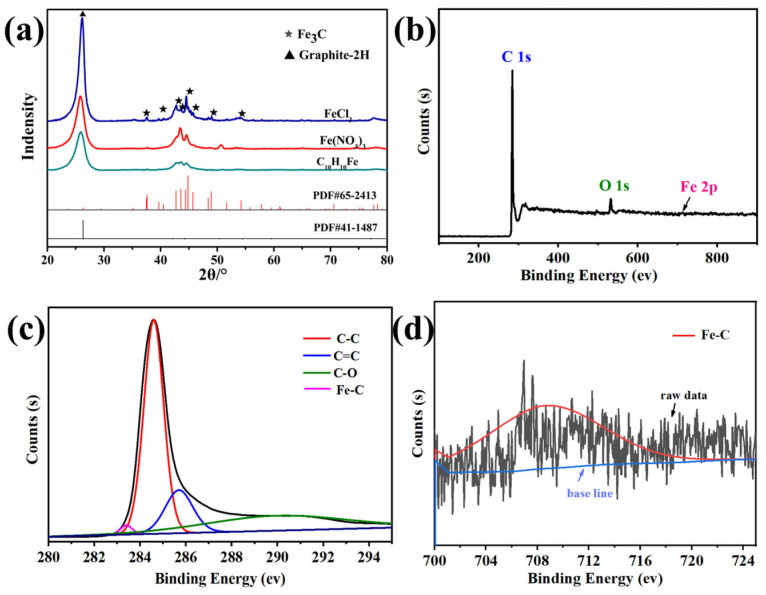
(**a**) XRD patterns of the porous carbon samples prepared with different anions. (**b**) XPS-survey spectra of FeCl_3_. (**c**) High-resolution XPS spectrum of C 1s. (**d**) High-resolution XPS spectrum of Fe 2p.

The X-ray diffraction (XRD) analysis of porous carbon samples synthesized with different anionic iron salts (FeCl_3_, Fe(NO_3_)_3_, and C_10_H_10_Fe) reveals consistent phase composition across all specimens, as illustrated in Figure 11a. All diffraction patterns exhibit characteristic peaks corresponding to the graphitic carbon phase and iron carbide phases. This observation indicates that different anionic (Cl⁻, NO_3_⁻, or organometallic ligands) do not significantly influence the final phase evolution during pyrolysis. The thermal decomposition pathways of the iron salts to similar intermediate products under high-temperature carbonization conditions, where the anion-derived volatile species (e.g., HCl, NO_X_, or hydrocarbons) are fully evolved, leaving metallic iron nanoparticles to catalyze graphitization and subsequently react with carbon to form thermodynamically stable iron carbides. The bonding characteristics and elemental composition of FeCl_3_ sample are further confirmed with XPS (Figure 11b–d). The near-surface composition determined by XPS (Figure 11b) indicated that the atomic contents of C, O and Fe in FeCl_3_ were 97, 2.5 and 0.5%, respectively, which matched the elemental mapping results obtained from the ICP-OES analysis discussed earlier. Figure 11c shows that the peaks at 284.5 eV, 285.9 eV and 289.3 eV for C 1s correspond to C–C, C=C of sp2 graphitic carbon and C–O bonds, respectively [32]. The dominant Fe–C peak (283.4 eV) corresponds to Fe_3_C. The Fe–C peak at 708.9 eV is attributed to Fe_3_C (Figure 11d).

### 3.4. The Joining of Silicon Carbide Parts

**Figure 12:**
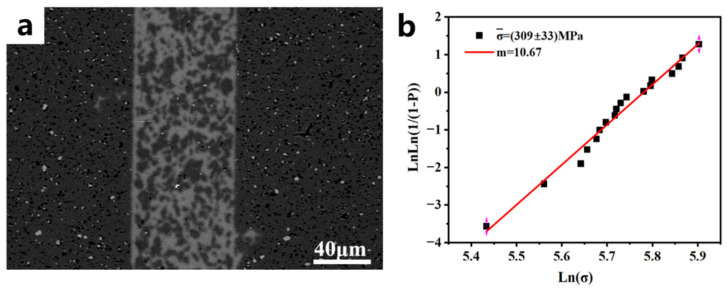
(**a**) Microstructure of the joint and (**b**) Flexural strengths and Weibull modulus of the joint.

The theoretical density of the porous carbon preform that can react completely with liquid silicon without silicon residue is 0.963 g·cm^−3^ [37]. In this study, a resin-based solution with added FeCl_3_ was selected for the subsequent joining experiment. After carbonization, the average pore diameter was 248 ± 29 nm, and the volume density of the porous carbon preform was 0.95 ± 0.02 g·cm^−3^, which was close to the theoretical density of the porous carbon preform. This structural feature provided an ideal transmission channel for the kinetics of the liquid silicon infiltration reaction. The FeCl_3_^−^ induced continuous meso-macro pore network effectively reduced the capillary resistance during the silicon melt infiltration process. The three-dimensional continuous pore structure maintained a stable chemical potential gradient at the reaction interface, thereby inhibiting the local enrichment of unreacted silicon. The microstructure of the joint of the joined sample is shown in Figure 12a. The joint structure was dense, without cracks, and there were no pores at the interlayer and interface. The results of the flexural strength and Weibull modulus of the joined specimens at room temperature are shown in Figure 12b. The flexural strength reached 309 ± 33 MPa, and the Weibull modulus reached 10.67. The high flexural strength and high Weibull modulus revealed the uniformity of the defect distribution in the connection layer, which was directly related to the uniformity of the spatial distribution of Fe^3^⁺ ions in the resin solution. The coordination effect of metal ions promoted the formation of a resin network with uniformly distributed pore structure characteristics, significantly improving the uniformity of SiC/Si distribution in the connection layer, which was manifested as a narrow dispersion characteristic of strength distribution at the macroscopic scale.

The thermal shock resistance of the joining samples was evaluated by testing their flexural strength after 30 cycles from room temperature to 450 °C. The flexural strength reached 318 ± 23 MPa after 30 cycles. The slight increase in flexural strength can be attributed to the formation of SiO_2_ after SiC oxidation, which effectively fills surface defects. This not only prevents further oxidation but also enhances the mechanical properties. Compared with the existing reaction joining systems (Table 5), this study successfully induces the formation of a three-dimensionally interconnected macroporous structure via metal salts during resin carbonization. This significantly enhances the molten silicon infiltration process, achieving both high joining strength and an impressive Weibull modulus of 10.67. The excellent stability and reliability of the joint further highlight its superior comprehensive mechanical properties.

**Figure 13:**
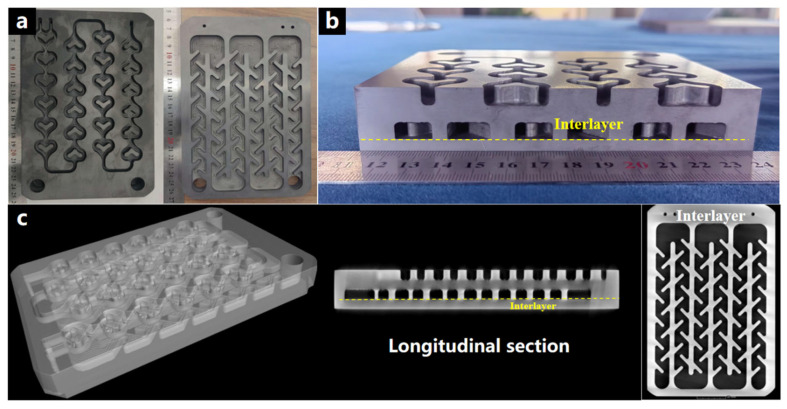
(**a**) Photos of SiC ceramic parts for joining (**b**) Sample profile photo after joining and (**c**) Industrial CT nondestructive testing.

This study conducted connection research on a large-sized 135 mm × 205 mm silicon carbide ceramic heat exchanger (Figure 13a). The cross-sectional morphology after connection (Figure 13b) shows that no typical pore defect band was observed at the connection interface. The industrial CT non-destructive testing three-dimensional reconstruction results (Figure 13c) further reveal the structural integrity of the connection body at the macroscopic scale. The isotropic gray-scale distribution feature indicates the high uniformity of the connection layer, which is attributed to the uniform distribution of the three-dimensional connected pore structure within the porous green body. During the connection thermodynamic process, the three-dimensional connected pores of the carbon skeleton regulate the local supersaturation of silicon vapor, ensuring the dynamic stability of the connection process of large-sized components. The process reliability demonstrated by this technical system validates the significant role of structured porous media in the field of ceramic connection. The three-dimensional connected pore network provides a penetration channel for liquid silicon, which provides a theoretical basis and engineering practice foundation for the development of connection technology for large-sized new ceramic matrix composite structures.

## 4. Conclusions

In summary, the pore structure of the porous carbon materials was successfully regulated by controlling the mass ratio of organic resin to solvent, the type of solvent, the valence state of metal ions, and the type of coexisting anions in the metal ion-resin-solvent system. As the molecular weight and viscosity of the solvent increased, the porosity of the porous carbon gradually decreased. When ethylene glycol was used as the solvent, the porosity reached a maximum of 52.9%. As the mass ratio of resin to solvent decreased, the porosity gradually increased. When the mass ratio was lower than 2:8, the sample could not form a carbon skeleton. The selection of metal ions (Fe^2+^ and Fe^3+^) significantly affected the formation mechanism of the pore structure. Fe^2+^ promoted the ortho-substitution of the phenolic structure of the resin, improved the resin polymerization, accelerated the phase separation process, and led to a higher porosity (i.e., 63.3%) with an average pore size of 190 ± 15 nm. In the preparation system of porous carbon materials, the anion coordination environment had a significant regulatory effect on the thermal decomposition kinetics of the metal precursor and the sol-gel phase separation process. Strong electrolyte metal salts (NO^3−^, Cl^−^) had a better pore-forming effect compared to metal organic compounds. Based on this study, a high-reliability connection of 135 mm × 205 mm large-sized silicon carbide ceramics was achieved using the resin solution. The connection layer was dense and defect-free, with a connection strength of 309 ± 33 MPa and a Weibull modulus of 10.67, verifying the important role of structured porous media in the field of large-sized ceramic connections.

## Figures and Tables

**Table 1:** Effect of solvent type on the pore structure of porous carbon.

Solvent Type	Melting Point /°C	Viscosity /mPa·s	Average Molecular Weight	Bulk Density (g·cm^−3^)	Apparent Porosity (%)	Materials Compositions (wt%)
EG	186.5	16	62.07	0.71 ± 0.03	52.9 ± 1.0	97.83 (C), 0.34 (Fe), 1.83 (O)
DEG	245.5	28	106.12	0.98 ± 0.02	34.7 ± 0.9	97.24 (C), 0.38 (Fe), 1.83 (O)
TEG	288.0	35	150.17	1.08 ± 0.04	28.1 ± 1.3	97.45 (C), 0.41 (Fe), 2.14 (O)
PEG200	>250	31	200.00	1.19 ± 0.03	21.2 ± 1.1	98.23 (C), 0.40 (Fe), 1.37 (O)
PEG400	>250	41	400.00	1.10 ± 0.02	16.5 ± 0.8	96.53 (C), 0.45 (Fe), 3.02 (O)

**Table 2:** Performance parameters of porous carbon materials prepared from resin mixtures with different PF/EG mass ratios.

Resin/Solvent	Apparent Porosity (%)	Bulk Density (g·cm^−3^)	Carbon Residue Rate/%	Materials Compositions (wt%)
7:3	34.2 ± 2.6	0.99 ± 0.02	34.83 ± 1.9	96.23 (C), 0.42 (Fe), 3.35 (O)
5:5	52.9 ± 3.2	0.71 ± 0.04	25.53 ± 2.1	97.83 (C), 0.34 (Fe), 1.83 (O)
1:2	66.5 ± 1.6	0.50 ± 0.03	19.48 ± 1.5	97.28 (C), 0.31 (Fe), 2.41 (O)
2:8	——	——	5.61 ± 0.7	95.14 (C), 0.21 (Fe), 4.65 (O)

**Table 3:** Specific structural parameters of the internal pore structure of porous carbon samples prepared using Fe ions with different valance states.

Sample	Apparent Porosity (%)	Average Aperture (nm)	Bulk Density (g·cm^−3^)	V_total_ (cm^3^·g^−1^)	Skeleton Density (g·cm^−3^)	Materials Compositions (wt%)
NoFe	25.6 ± 1.1	14 ± 5	1.18 ± 0.08	0.21 ± 0.07	1.55 ± 0.04	98.11 (C), 1.89 (O)
FeCl_2_	63.3 ± 1.7	190 ± 15	0.73 ± 0.01	0.86 ± 0.02	1.91 ± 0.05	97.83 (C), 0.34 (Fe), 1.83 (O)
FeCl_3_	50.9 ± 1.3	248 ± 29	0.95 ± 0.02	0.75 ± 0.03	1.94 ± 0.03	97.51 (C), 0.45 (Fe), 2.04 (O)

**Table 4:** Pore structure parameters of porous carbon materials prepared with different anions.

Sample	Apparent Porosity (%)	Average Aperture (nm)	Bulk Density (g·cm^−3^)	V_total_ (cm^3^·g^−1^)	Skeleton Density (g·cm^−3^)	Materials Compositions (wt%)
FeCl_3_	50.9 ± 1.3	248 ± 29	0.95 ± 0.02	0.75 ± 0.03	1.94 ± 0.03	97.51 (C), 0.45 (Fe), 2.04 (O)
Fe(NO_3_)_3_	51.4 ± 1.9	228 ± 21	0.97 ± 0.01	0.67 ± 0.03	1.89 ± 0.05	97.42 (C), 0.46 (Fe), 2.12 (O)
C_10_H_10_Fe	30.9 ± 1.9	8 ± 3	1.21 ± 0.02	0.24 ± 0.04	1.75 ± 0.02	97.51 (C), 0.45 (Fe), 2.04 (O)
no Fe	25.6 ± 1.1	14 ± 5	1.18 ± 0.08	0.21 ± 0.07	1.55 ± 0.04	98.11 (C), 1.89 (O)

**Table 5:** A comparison of the joining performance of SiC in other studies with that achieved in this work.

Joining Base Material	Interlayer Materials	Joining Condition	Joint Strength	Source
SiC-SiC	PF-FeCl_3_	Silicon powder, 1600 °C, vac	Flexural strength 309 MPa; Weibull modulus 10.67	This work
SiC-SiC	Parchment paper	1450~1550 °C, vac	Flexural strength 243~246 MPa	[38]
C_f_/SiC- C_f_/SiC	PF	Silicon powder, 1600 °C, vac	Flexural strength 203 ± 24 MPa	[39]
SiC_f_/SiC- SiC_f_/SiC	Si-Ti/SiC	Si-Ti infiltration, 1350 °C 2 h	/	[40]
additive-manufactured SiC	graphite paper	Silicon powder, 1550 °C 2 h	/	[16]
SiC-SiC	/	Spark Plasma Sintering (SPS) 1900 °C, 5 min, 60 MPa,	Flexural strength 193 ± 21 MPa	[41]

## Data Availability

The original contributions presented in this study are included in the article. Further inquiries can be directed to the corresponding authors.
